# Inpatient prescribing patterns of long-acting injectables and their oral or short-acting injectable equivalent formulations

**DOI:** 10.3389/fphar.2023.1140969

**Published:** 2023-05-22

**Authors:** Yifei Liu, Mark E. Patterson, Suman Sahil, Steven C. Stoner

**Affiliations:** ^1^ Division of Pharmacy Practice and Administration, University of Missouri-Kansas City School of Pharmacy, Kansas City, MO, United States; ^2^ Department of Biomedical and Health Informatics, University of Missouri-Kansas City School of Medicine, Kansas City, MO, United States

**Keywords:** long-acting injectable antipsychotic medications, prescribing patterns (physician), schizophrenia, schizoaffective disorder, bipolar disorder, real-world evidence

## Abstract

**Background:** Long-acting injectable (LAI) antipsychotics (APs) each have an oral equivalent formulation, while aripiprazole, olanzapine, and ziprasidone each also have a short-acting injectable (SAI) equivalent formulation. Inpatient prescribing patterns of LAIs and their oral/SAI equivalents are less characterized in populations other than Medicaid, Medicare, and Veterans Affairs populations. Mapping out inpatient prescribing patterns remains an important first step to ensure appropriate use of antipsychotics during this critical juncture of patient care prior to discharge. This study determined inpatient prescribing patterns of first- (FGA) and second-generation antipsychotic (SGA) LAIs and their oral/SAI formulations.

**Methods:** This was a large retrospective study using the Cerner Health Facts® database. Hospital admissions due to schizophrenia, schizoaffective disorder, or bipolar disorder from 2010 to 2016 were identified. AP utilization was defined as the proportion of inpatient stays during which at least 1 AP was administered to the total number of inpatient visits over the observed period. Descriptive analyses were used to determine prescribing patterns for APs. Chi-square tests were used to determine utilization differences across years.

**Results:** 94,989 encounters were identified. Encounters during which oral/SAI of SGA LAIs were administered were most common (n = 38,621, 41%). Encounters during which FGA LAIs or SGA LAIs were administered were the least common (n = 1,047, 1.1%). Prescribing patterns differed across years (*p* < 0.05) within the SGA LAI subgroup analysis (N = 6,014). Paliperidone palmitate (63%, N = 3,799) and risperidone (31%, N = 1,859) were the most frequently administered. Paliperidone palmitate utilization increased from 30% to 72% (*p* < 0.001), while risperidone utilization decreased from 70% to 18% (*p* < 0.001).

**Conclusions:** Compared with their oral or SAI formulations, LAIs were underutilized from 2010 to 2016. Among SGA LAIs, the prescribing patterns of paliperidone palmitate and risperidone changed significantly.

## Introduction

Schizophrenia and bipolar disorder are two of the costliest mental health diseases in the United States, accounting for approximately $281 (Schizophrenia and Psychosis Action Alliance, 2021) and $202 billion per year (Cloutier et al., 2018) in U.S. healthcare costs, respectively. Schizophrenia affects 0.8% (Schizophrenia and Psychosis Action Alliance, 2021), schizoaffective disorder affects 0.3% (Perälä et al., 2007; Olfson et al., 2009), and bipolar disorder affects 2.4% of the U.S. population (Merikangas et al., 2007). A sizable portion of these costs are driven by non-adherence to antipsychotics (APs) (Olivares et al., 2013), treatment interruptions that increase the risk of re-hospitalization (Weiden et al., 2004) and emergency psychiatric services (Hong et al., 2011).

Long-acting injectables (LAIs) include first- (FGA) and second-generation antipsychotic (SGA) LAIs. Before FGA and SGA LAIs were introduced to the market, they were preceded by their oral and short-acting injectable (SAI) equivalent formulations. Of note, ziprasidone is available in an oral formulation and as a SAI, but not as a LAI. Except for risperidone and paliperidone palmitate, each LAI also has a short-acting injectable equivalent formulation. LAIs are appropriate for patients with more severe illness, limited social supports, or at high-risk of relapse (Sajatovic et al., 2018), all of which are risk factors for medication non-adherence. Real-world studies illustrate that LAIs reduce hospitalization and non-adherence amongst Medicaid (Bera et al., 2013; Marcus et al., 2015; Pilon et al., 2017a; Pilon et al., 2017b), Medicare (Offord et al., 2013) and Veterans Affairs (VA) populations (Baser et al., 2015). A recent meta-analysis also illustrate that LAIs reduce the likelihood of non-adherence and hospitalization (Lin et al., 2021). Despite these promising findings in conjunction with a recent trend of increased LAI use, LAIs remain underutilized within those diagnosed with persistent psychiatric disorders (Rittmannsberger et al., 2017).

Although previous utilization studies provide evidence of underuse, large retrospective studies focus only on outpatient prescribing. Outpatient LAI use amongst Medicaid beneficiaries diagnosed with schizophrenia range from 10% for any LAI (Brown et al., 2014), to 3.1% and 3.8% for Risperdal, and FGA LAIs, respectively (Jackson et al., 2018). Outpatient LAI use amongst commercially insured beneficiaries with schizophrenia range from 6.9% to 9.3% for atypical and typical LAI, respectively (Fu et al., 2022). Inpatient prescribing studies are fewer in number with much smaller sample sizes. One inpatient study found that only 25%–33% of patients having clear indications for LAIs were prescribed LAIs prior to discharge (Kishimoto et al., 2017). Another study of 179 patients hospitalized for schizophrenia reported a 42% LAI utilization rate (Yee et al., 2021). Taken together, no studies to our knowledge leverage large retrospective databases to determine inpatient prescribing patterns within and among LAIs, oral APs, or SAIs.

Although SGA LAIs have emerged as treatment options with potential advantages including enhanced tolerability, dosing flexibility, and extended dosing time intervals, their prescribing patterns have not been widely reported in literature, as they are still relatively new medication administration technologies. Identifying gaps in inpatient prescribing patterns across a larger cross-section of APs is a critical step in achieving a better understanding of disparities, similarities, and differences within LAIs, oral APs, and SAIs, in turn informing better pharmacotherapy strategies prior to patients being discharged. Understanding prescribing patterns also has clinical merits, such as determining which SGA LAI is favored by prescribers.

Determining inpatient prescribing patterns provides insights into medication utilization during a critical period in which patients with schizophrenia are being stabilized, underscoring the need for studies measuring inpatient prescribing patterns. Furthermore, conducting these analyses using the Cerner Health Facts® Data representing hospitals across the US has the advantage of capturing real-world prescribing behaviors across a large dataset that would otherwise not be captured in randomized trials or smaller studies focused on inpatient prescribing.

## Objectives

Our primary objectives were to determine and describe FGA and SGA LAI and oral/SAI equivalent prescribing patterns within a sample of US patients hospitalized for schizophrenia, schizoaffective disorder, or bipolar disorder from 2010 to 2016. Our secondary objective was to identify SGA LAI prescribing pattern changes from 2010 to 2016.

## Methods

The Health Facts® database (Cerner Corp., Kansas City, MO) was used to examine prescribing patterns of APs for patients hospitalized for schizophrenia, schizoaffective disorder, or bipolar disorder between 2010 and 2016. The Cerner Health Facts® Data Warehouse includes records for over 64 million patients treated at over 863 hospitals and clinics throughout most states within the U.S. Currently, the database contains data from the inpatient setting on 11.4 million hospital inpatient stays and 22.9 million emergency department visits, and contains patient demographics, diagnosis, medications, and procedures. Only medications administered through hospital pharmacies are captured, such as National Drug Codes (NDCs), and the dates and times when drugs were dispensed.

The unit of analysis was an inpatient encounter. We included inpatient encounters (hospital stays or emergency department visits) for which the primary diagnosis was schizophrenia, schizoaffective disorder, or bipolar disorder, and during which at least one LAI or an oral/SAI equivalent formulation was prescribed. The diagnoses were based on ICD-9 and ICD-10 codes. ICD-9 codes were schizophrenia and schizoaffective disorder (ICD-9 Codes 295.0 to 295.9, including all the double digits), or bipolar disorder (ICD-9 Codes 296.0, 296.4 to 296.8, including all the double digits). ICD-10 codes were schizophrenia (ICD-10 Codes F20.0 to F20.9), schizoaffective disorder (ICD-10 Code F25), or bipolar disorder (ICD-10 Code F31).

Generic and brand-named FGA and SGA LAIs and their oral/SAI equivalent formulations were identified using NDCs. The Health Facts® database contained multiple NDCs corresponding to varying dosage forms for each of these generic and brand name drugs. FGA LAIs included haloperidol decanoate (Haldol®, Haldol Decanote®) and fluphenazine decanoate (Prolixin®). SGA LAIs included aripiprazole monohydrate (Abilify Maintena®), aripiprazole lauroxil (Aristada®), paliperidone palmitate (Invega Trinza®, Invega Sustenna®), risperidone (Risperdal Consta®), and olanzapine pamoate (Zyprexa Relprevv®). Of note, Risperidone (Perseris®) and aripiprazole lauroxil nanocrystal technology (Aristada Initio®) were not commercially available during the 6-year follow-up period of this study.

AP utilization consisted of inpatient encounters (hospital stays or emergency department visits) during which at least one LAI, oral AP, or SAI was dispensed. SAIs were included because agitated or acutely psychotic patients often receive a short-acting intramuscular form of the medication they eventually will be administered orally during their hospitalization. Including SAIs enabled us to capture total use of all different formulations of the AP class. We divided the inpatient encounters into fifteen mutually exclusive medication categories corresponding to fifteen different combinations of prescribing patterns (Appendix). Descriptive analyses were conducted for these fifteen inpatient prescribing patterns. For each prescribing pattern, Chi-square tests were used to examine whether the pattern differed across years.

In the sub-group analysis, we examined inpatient encounters during which any of the SGA LAIs was dispensed. Descriptive analyses were used to determine utilization rates within the 6,014 encounters. For each year, the utilization rate was calculated as the number of encounters associated with a particular SGA LAI divided by the number of encounters associated with all SGA LAIs. For each SGA LAI, Chi-square tests were used to determine if the utilization rates differed across years. All analyses were completed using IBM SPSS Statistics Version 25 (IBM Corp., Armonk, NY), and this study was under a long-standing IRB approval for all Health Facts® projects at our institution.

## Results

A total of 94,989 encounters were identified, including 80,648 hospital stays (84.9%) and 14,341 emergency department visits (14.9%). FGA oral/SAI formulations were administered in 88.6% of the inpatient encounters, and SGA oral/SAI formulations were administered in 40.7% of encounters (Table 1). LAIs as monotherapy were administered in 1.1% of inpatient encounters, while LAI and oral/SAI equivalents were administered concomitantly in 10.3% of inpatient encounters. In addition, FGA LAIs and SGA LAIs were only administered as monotherapy in 0.52% and 0.58% of inpatient encounters, respectively.

**TABLE 1:** Inpatient prescribing patterns of APs 2010–2016 (N = 94,989).

Inpatient prescribing patterns	N (%)
*LAI only*	
FGA LAI only	494 (0.52)
SGA LAI only	553 (0.58)
FGA and SGA LAI only	0 (0)
**Subtotal**	**1,047 (1.1)**
*Oral/SAI formulations only*	
FGA oral/SAI only	22,926 (24.1)
SGA oral/SAI only	38,621 (40.7)
FGA and SGA oral/SAI only	22,644 (23.8)
**Subtotal**	**84,191 (88.6)**
*Concurrent LAI and oral/SAI equivalent*	
FGA oral/SAI and FGA LAI	2,496 (2.6)
SGA oral/SAI and SGA LAI	2,148 (2.3)
FGA oral/SAI and SGA LAI	481 (0.51)
SGA oral/SAI and FGA LAI	274 (0.29)
FGA oral/SAI, FGA LAI, and SGA oral/SAI	1,587 (1.7)
FGA oral/SAI, FGA LAI, and SGA LAI^a^	18 (0.02)
SGA oral/SAI, SGA LAI, and FGA oral/SAI	2,615 (2.75)
SGA oral/SAI, SGA LAI, and FGA LAI^a^	15 (0.02)
FGA oral/SAI, SGA oral/SAI, FGA LAI, and SGA LAI^a^	117 (0.12)
**Subtotal**	**9,751 (10.3)**
**Total**	**94,989 (100)**

Notes.

FGA, LAIs: haloperidol decanoate (Haldol®, Haldol Decanoate®) and fluphenazine decanoate (Prolixin®).

SGA, LAIs: aripiprazole monohydrate (Abilify Maintena®), aripiprazole lauroxil (Aristada®), paliperidone palmitate (Invega Trinza®, Invega Sustenna®), risperidone (Risperdal Consta®), and olanzapine pamoate (Zyprexa Relprevv®).

FGA, oral equivalents of FGA, LAIs.

SGA, oral^:^ oral equivalents of SGA, generation LAIs.

^a^ Pattern did not differ significantly across years.

**FIGURE 1:**
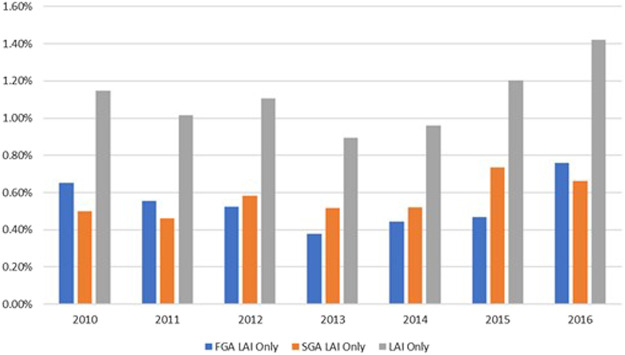
Trends of Prescribing Patterns for FGA LAI, SGA LAI, and LAI monotherapy 2010–2016.

Figure 1 displays the trends of FGA LAI, SGA LAI, and LAI monotherapy. Overall, the utilization of FGA LAI and LAI monotherapy increased between 2013 and 2016. The utilization of SGA LAI monotherapy increased between 2013 and 2015. Chi-square tests for each prescribing pattern across years were statistically significant (*p* < 0.05), except the following three categories.

1. The concurrent use of oral/SAI formulations of FGA LAIs, FGA LAIs, and SGA LAIs.
2. The concurrent use of oral/SAI formulations of SGA LAIs, SGA LAIs, and FGA LAIs.
3. The concurrent use of oral/SAI formulations of FGA LAIs, oral/SAI formulations of SGA LAIs, FGA LAIs, and SGA LAIs.

Sixty-three percent of encounters were associated with patients who were Caucasian, and 28% were associated with patients who were African American (Table 2). Fifty-six percent of encounters were associated with male patients, and 69% were associated with single patients. The average age across encounters was 44.8 years old (±15.5), and the average length of stay was 8.7 days (±13.7). In addition, 83% of encounters were associated with urban hospitals, and 91% were associated with acute hospitals (Table 2).

**TABLE 2:** Patient demographics and facility characteristics associated with encounters (N = 94,989).

Variables	Frequency	Percent
Patient demographics		
Race		
Caucasian	60,008	63.2
African American	26,562	28
Asian or Pacific Islander	1,701	1.7
Hispanic	1,310	1.4
Native American	1,028	1.1
Other	4,380	4.6
Total	94,989	100
Gender		
Male	52,812	55.6
Female	42,177	44.4
Total	94,989	100
Marital Status		
Single	65,638	69.1
Divorced	11,712	12.3
Married	11,281	11.9
Widowed	3,565	3.8
Other	2,793	2.9
Total	94,989	100
Facility characteristics		
Urban vs. Rural		
Urban	78,976	83.1
Rural	16,013	16.9
Total	94,989	100.0
Acute vs. Non-Acute		
Acute	86,063	90.6
Non-Acute	8,926	9.4
Total	94,989	100.0

**FIGURE 2:**
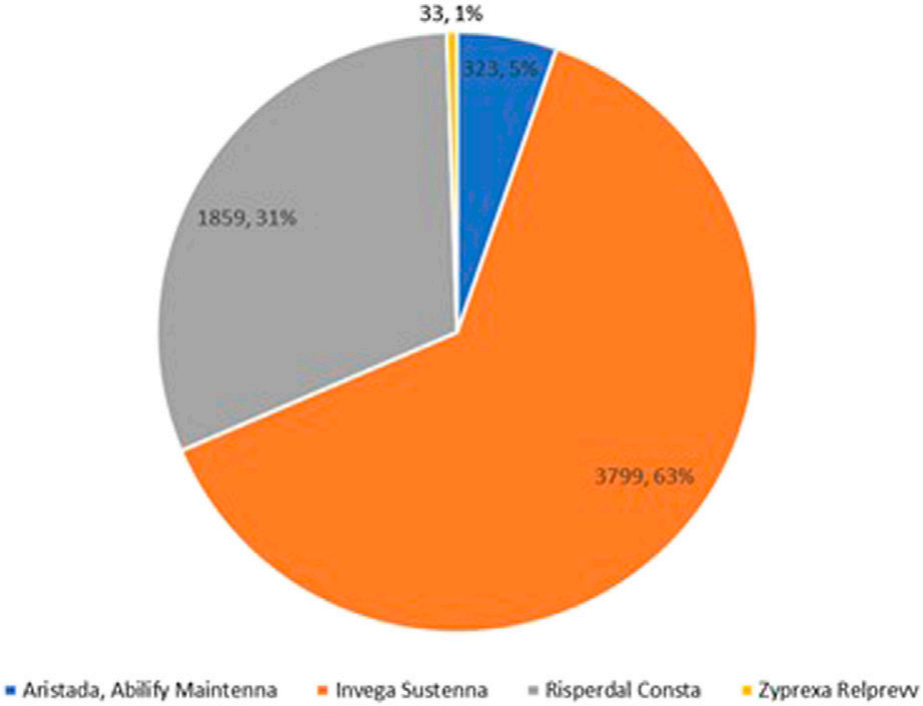
Proportion of medications within the subgroup of SGA LAIs 2010–2016 (N = 6,014).

**FIGURE 3:**
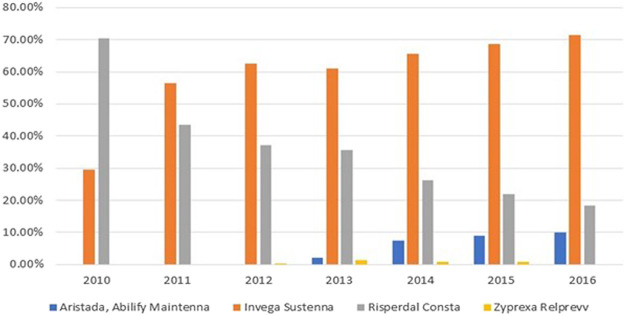
Year-specific utilization rates at the admission level for brand-named SGA LAIs.

In the sub-group analysis of SGA LAIs, 6,014 encounters were identified. Of note, the identification method was different from the method used in Table 1. For example, if an encounter was associated with both paliperidone palmitate and olanzapine pamoate, it was counted as two (one for paliperidone palmitate and one for olanzapine pamoate) but counted as one in the category of “SGA LAI only” in Table 1. As shown in Figure 2, Aripiprazole was administered in 5% (N = 323) of encounters, paliperidone palmitate was administered in 63% (N = 3,799) of encounters, risperidone was administered in 31% (N = 1,859) of encounters, and olanzapine was administered in less than 1% (N = 33) of encounters. Aripiprazole monohydrate (Abilify Maintena®) and aripiprazole lauroxil (Aristada®) were combined as one category of aripiprazole due to only four encounters related to Aristada®. Breaking these 6,014 encounters out across 7 years revealed paliperidone palmitate utilization increased from 29.6% to 71.6% (*p p*< 0.001), while risperidone decreased from 70.4% to 18.4% (*p* < 0.001) (Figure 3; Table 3).

**TABLE 3:** Year-specific utilization rates for SGA LAIs (N = 6,014).

	Overall	2010	2011	2012	2013	2014	2015	2016	*p*-value
SGA LAIs	N (col %)	N (col %)	N (col %)	N (col %)	N (col %)	N (col %)	N (col %)	N (col %)	
Aripiprazole	323 (5.4)	0 (0)	0 (0)	0 (0)	21 (2.1)	86 (7.5)	113 (8.9)	103 (10)	<0.0001
Paliperidone palmitate	3799 (63.2)	108 (29.6)	286 (56.5)	451 (62.5)	600 (61.1)	750 (65.5)	869 (68.5)	735 (71.6)	<0.0001
Risperidone	1859 (30.9)	257 (70.4)	220 (43.5)	267 (37)	349 (35.5)	300 (26.2)	277 (21.9)	189 (18.4)	<0.0001
Olanzapine pamoate	33 (0.5)	0 (0)	0 (0)	3 (0.4)	12 (1.2)	9 (0.8)	9 (0.7)	0 (0)	0.003
Total	6,014	365	506	721	982	1,145	1,268	1,027	

## Discussion

According to our knowledge, this was the first study to utilize a large nationwide sample to examine inpatient prescribing patterns of LAIs and concomitant oral/SAI APs for schizophrenia, schizoaffective disorder, or bipolar disorder. Our findings of low inpatient LAI utilization are consistent with previous studies conducted in smaller settings (Kishimoto et al., 2017; Yee et al., 2021). Previous studies determining inpatient LAI prescribing patterns had smaller sample sizes and focused on single sites such as psychiatric hospitals. In addition, for each of fifteen prescribing patterns, this study performed detailed comparisons across years, whereas previous studies only included certain APs. Moreover, this study compared the utilization rates among SGA LAIs. The Chi-square results for each prescribing pattern across years demonstrated that LAIs were underutilized. Figure 1 shows that paliperidone palmitate (63%) and risperidone (31%) were the two primary SGA LAIs utilized between 2010 and 2016. Furthermore, Figure 2 with Chi-square test results demonstrate a trend of increasing utilization for paliperidone palmitate since 2010, and for aripiprazole since 2013; and a trend of decreasing utilization for risperidone since 2010.

Our analysis reported lower LAI inpatient prescribing rates (e.g., 1.1% for LAI monotherapy, and 10.3% for concurrent use of LAI and oral/SAI formulation), compared to previous studies. Kishimoto et al. (2017) found LAIs were prescribed in 25%–33% of patients admitted to a 208-bed psychiatric hospital. Yee et al. (2021) found LAIs were prescribed in 42% of hospitalized patients participating in a community hospital-based community treatment program. The higher prescribing rates observed in these studies were potentially attributed to the fact they included individuals with a history of hospitalization due to AP non-adherence (Kishimoto et al., 2017) or individuals who experience the most intractable symptoms or level of dysfunction (Yee et al., 2021). The lower utilization rates in our study could be due to a host of differences in patient preferences, clinician knowledge and attitudes, coverage policies, or pharmaceutical company promotions - all of which have been shown to impact utilization trends in schizophrenic patients (Horvitz-Lennon et al., 2009). For example, familiarity with treatment guidelines and frequent contact with pharmaceutical representatives influence the physician prescribing behaviors of SGA LAIs (Arbuckle et al., 2008). The higher utilization rates in psychiatric hospitals admitted patients with more severe conditions and had providers who were intimately familiar with treatment guidelines, compared to our sample of patients at primarily general acute care facilities.

For SGA LAIs, we found 4% (3,799/94,989), 2% (1,859/94,989) and 0.3% (323/94,989) of hospitalizations had at least one administration of paliperidone, risperidone, and aripiprazole (Abilify Maintena® and Aristada® combined), respectively. In contrast, Yee et al. found 14%, 17% and 9% for paliperidone, risperidone, and aripiprazole (Abilify Maintena® only), respectively (Yee et al., 2021). Because Yee et al. conducted their study in 2018, when SGA LAIs became more widely available, and they focused on a designated clinical program in which patients might have more severe conditions, it is reasonable that the utilization rates were higher than our findings. However, the advantage of our study is offering longitudinal perspectives for a variety of prescribing patterns across healthcare facilities. As mentioned earlier, two noteworthy trends we observed were the upward and downward utilization trends of paliperidone and risperidone, respectively between 2010 and 2016. The proportion of encounters during which paliperidone was administered increased from 29.6% to 71.6%, yet the proportion of encounters during which risperidone was administered decreased from 70.4% to 21.9%. This reversed trend can partly be explained by prescribers preferring paliperidone over risperidone due to dosing interval extensions or improved tolerability. For example, patients initiated on paliperidone palmitate (Invega Sustenna®) have a longer injection cycle of 1 month which could later be extended to 3 months by switching to Invega Trinza®.

Despite these increasing trends over time, our results still affirm LAI underutilization within inpatient settings between 2010 and 2016. Underutilization can result in negative health outcomes by reducing symptom control, worsening symptoms, increasing hospitalization or readmission, and increasing healthcare expenditures related to non-adherence and associated psychiatric decompensation (Lacro et al., 2002; Nosé et al., 2003). Examining prescribing patterns of APs in the inpatient setting is the first step to address issues related to underutilization. The variation in prescribing patterns of APs and specifically SGA LAIs indicates disparities in medication utilization. The variation is clinically relevant because discharging patients on appropriate regimens would reduce readmissions and improve health outcomes. The variation could also lead to hypotheses about what patient, provider, or organizational factors would drive the differences. Therefore, future studies should focus on two directions: 1) exploring the relationship between prescribing patterns and patients’ health outcomes, especially how the underutilization of LAIs impacts patients’ health outcomes; and 2) identifying patient, provider, or organizational factors affecting the disparities of medication utilization or health outcomes. Furthermore, future studies could compare costs and quality of life measures between FGA LAIs and SGA LAIs, or among prescribing patterns of APs. If differences in quality of life were minimal, and differences in costs were significant, results would have important implications for prescribing behaviors and clinical recommendations.

This study had three limitations. First, the results are generalizable to only the types of facilities included in Health Facts® database and associated with Cerner Corp. Second, given the absence of data, we were not able to capture prescribing patterns in outpatient prescribing patterns, which also provide important context of prescribing patterns outside the hospital stay. Despite this, examining solely inpatient prescribing enables us to focus on a population of patients with more severe disease compared to those represented in outpatient clinics. Third, we were not able to measure prescriber-specific factors such as attitude or behavior, because specific providers were not identifiable in Health Facts® database. Despite these limitations, this is the first known study to using a large dataset of inpatient prescribing to evaluate the LAI prescribing patterns amongst US hospitals. The size of dataset provides results that are more generalizable compared to previous inpatient utilization studies focused on smaller sample sizes and fewer study sites. Furthermore, the detailed time and date stamps for medication administration in combination with the substantial number of hospitals represented within the HealthFacts® database offers robust “real-world” data to accurately capture physician prescribing patterns across a large representative sample of US hospitals.

## Conclusion

Our study reported fifteen inpatient prescribing patterns of APs for schizophrenia, schizoaffective disorder, or bipolar disorder from 2010 to 2016. Compared with their oral/SAI formulations, LAIs were underutilized at the time of acute mental health crisis and acute hospitalizations. In addition, among SGA LAIs, the prescribing patterns of paliperidone palmitate and risperidone changed significantly. Further study is needed to explore the extent to which patient, provider, or organizational factors are associated with FGA or SGA LAI utilization, and in turn, the extent to which higher LAI utilization is associated with lower incidence of emergency room visits or hospitalizations.

## Data Availability

The data analyzed in this study is subject to the following licenses/restrictions: Health Facts is a proprietary dataset only available to entities who have a data use agreement established with Cerner.

## Appendix: Classification method of medications

LAI only:

1. First-generation antipsychotic (FGA) LAIs
2. Second-generation antipsychotic (SGA) LAIs
3. Concurrent FGA and SGA LAIs

Oral AP/SAI Formulations only:

4. Oral AP/SAI formulations of FGA LAIs
5. Oral/SAI formulations of SGA LAIs
6. Concurrent oral/SAI formulations of FGA and SGA LAIs

Concurrent use of LAI and oral/SAI equivalents:

7. Oral/SAI formulations of FGA LAIs, and FGA LAIs
8. Oral/SAI formulations of SGA LAIs, and SGA LAIs
9. Oral/SAI formulations of SGA LAI APs, and SGA LAIs
10. Oral/SAI formulations of SGA LAIs, and FGA LAIs
11. Oral/SAI formulations of FGA LAIs, FGA LAIs, and oral/SAI formulations of SGA LAIs
12. Oral/SAI formulations of FGA LAIs, FGA LAIs, and SGA LAI APs
13. Oral/SAI formulations of SGA LAIs, SGA LAIs, and oral/SAI formulations of FGA LAIs
14. Oral/SAI formulations of SGA LAIs, SGA LAIs, and FGA LAIs
15. Oral/SAI formulations of FGA LAIs, oral/SAI formulations of SGA LAIs, FGA LAIs, and SGA LAIs
